# Autophagy inhibition improves the targeted radionuclide therapy efficacy of ^131^I-FAP-2286 in pancreatic cancer xenografts

**DOI:** 10.1186/s12967-024-04958-6

**Published:** 2024-02-15

**Authors:** Xingyu Liu, Danni Li, Tianbao Ma, Xiu Luo, Ye Peng, Tao Wang, Changjing Zuo, Jianming Cai

**Affiliations:** 1 School of Public Health and Management, Wenzhou Medical University, Wenzhou, 325035 Zhejiang China; 2 Department of Nuclear Medicine, The First Affiliated Hospital of Naval Medical University, Shanghai, 200433 China; 3 Department of Radiation Medicine, Faculty of Naval Medicine, Naval Medical University, Shanghai, 200433 China

**Keywords:** Pancreatic cancer, Autophagy, Targeted radionuclide therapy, FAP-2286

## Abstract

**Purposes:**

Radiotherapy can induce tumor cell autophagy, which might impair the antitumoral effect. This study aims to investigate the effect of autophagy inhibition on the targeted radionuclide therapy (TRT) efficacy of ^131^I-FAP-2286 in pancreatic cancer.

**Methods:**

Human pancreatic cancer PANC-1 cells were exposed to ^131^I-FAP-2286 radiotherapy alone or with the autophagy inhibitor 3-MA. The autophagy level and proliferative activity of PANC-1 cells were analyzed. The pancreatic cancer xenograft-bearing nude mice were established by the co-injection of PANC-1 cells and pancreatic cancer-associated fibroblasts (CAFs), and then were randomly divided into four groups and treated with saline (control group), 3-MA, ^131^I-FAP-2286 and ^131^I-FAP-2286 + 3-MA, respectively. SPECT/CT imaging was performed to evaluate the bio-distribution of ^131^I-FAP-2286 in pancreatic cancer-bearing mice. The therapeutic effect of tumor was evaluated by ^18^F-FDG PET/CT imaging, tumor volume measurements, and the hematoxylin and eosin (H&E) staining, and immunohistochemical staining assay of tumor tissues.

**Results:**

^131^I-FAP-2286 inhibited proliferation and increased the autophagy level of PANC-1 cells in a dose-dependent manner. 3-MA promoted ^131^I-FAP-2286-induced apoptosis of PANC-1 cells via suppressing autophagy. SPECT/CT imaging of pancreatic cancer xenograft-bearing nude mice showed that ^131^I-FAP-2286 can target the tumor effectively. According to ^18^F-FDG PET/CT imaging, the tumor growth curves and immunohistochemical analysis, ^131^I-FAP-2286 TRT was capable of suppressing the growth of pancreatic tumor accompanying with autophagy induction, but the addition of 3-MA enabled ^131^I-FAP-2286 to achieve a better therapeutic effect along with the autophagy inhibition. In addition, 3-MA alone did not inhibit tumor growth.

**Conclusions:**

^131^I-FAP-2286 exposure induces the protective autophagy of pancreatic cancer cells, and the application of autophagy inhibitor is capable of enhancing the TRT therapeutic effect.

**Supplementary Information:**

The online version contains supplementary material available at 10.1186/s12967-024-04958-6.

## Introduction

Pancreatic ductal adenocarcinoma (PDAC) remains the tumor with the poorest prognosis in the digestive tract, with a 5-year survival rate less than 10%, and is predicted to become the second leading cause of cancer-related death in western countries by 2030 [1]. Although surgical resection currently represents the best opportunity for cure and long-term survival, only up to 20% of patients present at a tumor stage suitable for resection [2]. Given that most of PDAC are diagnosed at a late stage with regional metastases and/or distant metastases, finding the proper treatment method is necessary to improve the dismal prognosis [3].

As a special form of radiotherapy, targeted radionuclide therapy (TRT) of tumor is based on the use of radiolabeled peptides or antibodies, which serve as the molecular carriers of radionuclides and bind to the overexpressed receptors or specific antigens in tumor cells [4]. Unlike external beam therapy, TRT allows the targeted radiopharmaceuticals delivery to the clinically diagnosed tumor site as well as the metastasized tumor cells, thus benefiting patients with late-stage and metastatic disease [5]. Therefore, TRT offers a new therapeutic option for PDAC. Some clinical trials of pancreatic cancer treatment have demonstrated the safety and potential efficacy of TRT, yet the therapeutic effect still need to be improved [6].

Fibroblast activation protein (FAP) is specifically overexpressed in pancreatic cancer-associated fibroblasts (CAFs), which are the major cell component of the tumor microenvironment in pancreatic cancer [7]. This selective expression provides a potential therapeutic target for TRT. In fact, radionuclide-labeled FAP inhibitors (FAPIs) have been developed for the targeted imaging or therapy of various types of cancers [8–10]. However, FAPIs with a short tumor retention time are unfavorable for TRT [11, 12]. FAP-2286 is a FAP-binding cyclic peptide, and exhibits prolonged tumor retention, which is an important prerequisite for TRT [13–15]. ^131^I with a relative long half-life of 8 days is a clinically used therapeutic radionuclide, allowing for prolonging radiation exposure and matching the tumor retention capabilities of FAP-2286 [16]. Based on these properties, ^131^I-labeled FAP-2286 may serve as a good TRT agent for pancreatic cancer, and certainly, strategies to further improve the therapeutic effect is warranted.

Generally, radiotherapy can cause cancer cell death through intrinsic, autonomous modalities directly triggered in the irradiated cells (e.g., apoptosis, autophagic cell death, mitotic catastrophe), or extrinsic, non-cell autonomous modalities mediated by the microenvironment or immune system (e.g., senescence, immunogenic cell death). However, the tumor cells also survive via the activation of the survival pathways, such as DNA damage repair, cell cycle arrest and autophagy, which allow cells to endure damage [17]. Autophagy of cancer cells is frequently observed during radiotherapy [18, 19]. Interestingly, pancreatic CAFs also has the ability to suppress the radiotherapy effect via promoting the autophagy of the irradiated pancreatic cancer cells [20, 21]. Therefore, it is desired to investigate whether ^131^I-FAP-2286 TRT affects the autophagy of pancreatic cancer cells, and whether the autophagy inhibitors help improve the therapeutic efficacy of TRT.

In this study, ^131^I-labeled FAP-2286-Tyr, abbreviate as ^131^I-FAP-2286, was developed for the TRT in pancreatic cancer. After confirming that ^131^I-FAP-2286 exposure can cause PANC-1 cells autophagy, the effect of autophagy inhibition on the in vitro and in vivo anti-tumor effect of ^131^I-FAP-2286 were assessed. Our present study suggests that autophagy inhibition can improve the TRT efficacy of ^131^I-FAP-2286 in pancreatic cancer.

## Materials and methods

### Materials and reagents

^131^I-NaI and ^18^F-FDG were provided by Shanghai Atom Kexing Pharmaceutical Co., Ltd. (Shanghai, China). DMEM, PBS, fetal bovine serum (FBS), 100 U/mL penicillin and 100 μg/mL streptomycin were purchased from Gibco-Thermo Fisher (Waltham, MA, USA). 1,3,4,6-Tetrachloro-3α,6α-diphenylglycouril (Iodogen) was purchased from Sigma Chemical (St. Louis, MO, USA). Polyclonal antibodies against LC3B, p62, β-actin and horseradish peroxidase-conjugated secondary antibodies were purchased from Cell Signaling Technology (Beverly, MA, USA). Autophagy inhibitor 3-Methyladenine (3-MA) was obtained from MCE company (Shanghai, China). Cell counting kit-8 (CCK-8) and Annexin V–FITC and propidium iodide (Annexin V/PI) apoptosis detection kit were purchased from Beyotime Institute of Biotechnology (Jiangsu, China). FAP-2286-Tyr was provided by Nice-labeling Biotechnology Co., Ltd. (Shanghai, China).

### The synthesis and in vitro stability of ^131^I-labeled FAP-2286-Tyr

For labeling FAP-2286-Tyr with ^131^I, a simple Iodogen method was used. In brief, 20 μL ^131^I-NaI solution (9.5 MBq/μL) and 100 μL PBS buffer containing 20 μg FAP-2286-Tyr were added into the Iodogen tube pre-coated with 50 μg Iodogen orderly. Iodogen tube was placed on vortex mixer at room temperature for 10 min, and then the developed ^131^I-FAP-2286 in the supernatant liquid was removed from the reaction tube.

The labeling rate of ^131^I-FAP-2286 was tested using radio instant thin-layer chromatography (radio-iTLC) method in which Whatman chromatographic strip paper and 0.9% NaCl (normal saline) was used as the stationary phase and mobile phase, respectively. The in vitro stability test was performed by mixing the labeled product with PBS, 10% FBS or normal saline, respectively, at 4 °C and 37 °C, and incubating for 1, 2, 4, 8, 12 and 24 h before determining the radiochemical purity.

### Cell culture

The human pancreatic carcinoma cells-1 (PANC-1) were obtained from Qui Cell Biology, Co., Ltd (Shanghai, China). Cells were cultured in DMEM supplemented with 10% FBS, 2 mM l-glutamine, 100 U/mL penicillin and 100 μg/mL streptomycin at 37 °C with 5% CO_2_. The pancreatic CAFs, derived from human surgical pancreatic cancer tissue with a doubling time of 3 days, were obtained from Kanglang Biology, Co., Ltd (Shanghai, China).

### Cell viability assays

PANC-1 cells with a volume of 200 μL containing 8 × 10^3^ cells were seeded into each well of 96-well plates. These cells were allowed to grow adherently for 24 h. The cells were treated with various doses of ^131^I-FAP-2286 (0, 3.7, 9.25, 18.5, and 37 MBq/mL) or different treatment groups including control (DMEM), 3-MA (5 μg/mL), ^131^I-FAP-2286 (9.25 MBq/mL) and ^131^I-FAP-2286 (9.25 MBq/mL) in combination with 3-MA (5 μg/mL) treatment. Following a 24-h incubation period, 10 μL of CCK-8 solution (5 mg/mL) was introduced to each well. After an additional 2-h incubation, the optical density at 450 nm (OD_450_) was quantified using a spectrophotometric microplate reader (Multiskan FC, Thermo). The cell viability (%) was calculated by normalizing the OD_450_ values to the untreated wells.

### Western blot analysis

The expression of p62, a classical marker of autophagy, in PANC-1 cells exposed to ^131^I-FAP-2286, was measured by western blotting. To assess the influence of CAFs on the autophagy of PANC-1 induced by ^131^I-FAP-2286, the cell co-culture experiment was performed in a 6-well plate with 3 μm pore size transwell inserts, where PANC-1 cells and CAFs were seeded into the bottom wells and the upper inserts, respectively. After a 24-h culture with ^131^I-FAP-2286, p62 expression in PANC-1 was detected. After receiving the corresponding treatments, the cells were lysed using RIPA lysis buffer in the presence of protease and phosphatase inhibitors (Minitab, Roche). The lysates were sonicated on ice and then centrifuged at 12,000 rpm at 4 °C for 10 min. The supernatants were collected for the protein concentration detection, and then were exposed to 12% SDS–polyacrylamide gel electrophoresis. The separated proteins were transferred to nitrocellulose membranes, followed by being blocked with Odyssey blocking buffer (LI-COR) in PBS containing 1% Tween-20. Primary antibodies of p62 and β-actin were incubated in blocking buffer at 4 °C overnight, and then were detected using HRP-conjugated secondary antibody. The photos of the target protein bands were captured using LI-COR Odyssey protein imaging system and quantified by Image J software.

### Immunofluorescence (IF)

PANC-1 cells were cultured on coverslips in 6-well plates, and then were incubated for 24 h after receiving the responding treatments. After being fixed for 5 min at − 20 °C with ice-cold methanol, cells were blocked for 1 h at room temperature with 5% BSA, followed by incubation with primary antibodies (LC3II, p62) overnight at 4 °C. Following that, the cells were rinsed with PBS and incubated for 1 h at room temperature with Alexa Fluor 488-conjugated secondary antibodies. Nuclei were stained with DAPI for 10 min at room temperature. The cells on the slides were observed under a fluorescence microscope (magnification, ×100; BX53, Olympus Corporation).

### Transmission electron microscopy (TEM)

PANC-1 cells were collected and centrifuged at 1200 rpm for 5 min. The supernatant was discarded, and then the cells were quickly fixed with electron microscope fixation solution at 4 °C for 2 h. After this, the cell samples were transported to Servicebio Biotechnology Co., Ltd (Wuhan, China), and the sample preparation process for the conventional TEM was carried out. Finally, 60 nm ultrathin sections were obtained and observed using Hitachi-7500 transmission electron microscope (Hitachi, Japan).

### Calcein-AM/PI assay

After treatment, PANC-1 cells were added with Calcein-AM/PI staining solution and incubated at 37 °C for 20 min following the manufacturer instructions. Under the fluorescence microscope, the living cells and dead cells were identified as green fluorescence and red fluorescence, respectively.

### Apoptosis detection

Cell apoptosis was determined using an Annexin-V-FITC/propidium iodide (PI) apoptosis kit. PANC-1 cells upon different treatments were cultured in 6-well culture plates, Cells were collected and washed twice with ice-cold PBS before double staining with Annexin V-FITC/PI after 24 h. Apoptosis detection was performed in a flow cytometer and the results were analyzed using the CellQuest Pro (IVD) software (BD, Bioscience).

### ^131^I-FAP-2286 TRT of pancreatic cancer xenograft-bearing nude mice

All animal experiments were approved by the Ethics Committee of Naval Medical University, and were conducted with the guidance of ethical principles governing animal welfare, rearing, and experimentation. Female nude mice (age, 4 weeks) were purchased from Vital River Laboratory Animal Technology Co., Ltd, and were raised under specific pathogen-free (SPF) conditions. For the establishment of subcutaneous xenograft pancreatic cancer model in nude mice, 100 μL of cell suspension of PANC-1 (5 × 10^6^) with CAFs (5 × 10^6^) or PANC-1 (5 × 10^6^) alone were injected into the right upper limb, respectively. The size of tumor was measured every other day. When the tumor volume reached about 60 mm^3^, pancreatic cancer xenograft-bearing nude mice were chosen for treatment experiments. The mice were randomly divided into four treatment groups: Control (saline), 3-MA, ^131^I-FAP-2286 and ^131^I-FAP-2286 combined with 3-MA. For 3-MA group, mice received daily intraperitoneal injection of 3-MA (15 mg/kg/d) for 14 d. For ^131^I-FAP-2286 group, mice were intravenously injected with ^131^I-FAP-2286 (9.25 MBq per mouse). In ^131^I-FAP-2286 combined with 3-MA group, 3-MA was administered at 1 d after the injection of ^131^I-FAP-2286. The tumor volume was calculated by the equation: tumor volume (mm^3^) = (length × width^2^)/2. After 2 weeks, the mice were sacrificed by overdose anesthesia, and the tumor tissues were harvested and stored in 4% paraformaldehyde fix solution for subsequent H&E staining histopathology, and FAP, α-SMA, Ki-67, p62 and LC3B immunohistochemistry assay (Servicebio, Wuhan, China).

### SPECT/CT imaging of pancreatic cancer xenograft-bearing nude mice

In this experiment, free-[^131^I] or ^131^I-FAP-2286 (9.25 MBq/200 μL) was injected into pancreatic cancer xenograft-bearing nude mice (PANC-1 alone, or PANC-1 + CAFs co-injection) via tail vein, and then SPECT/CT imaging was performed at different time points post injection. For tomography with high-energy high-resolution collimator, matrix: 64 × 64; zoom: 2; energy peak: 35 keV; window width: 20%; frame size: 60 s/frames; total acquisition of 32 frames. For CT, tube voltage: 130 kV; tube current: 35 mA; pitch: 1.0; reconstructed layer thickness: 1 mm. 3D regions of interest (ROIs) were drawn over the whole-body, brain, thyroid, lungs, heart, liver, spleen, intestine, muscle and tumor on decay-corrected whole-body images to obtain the total counts and volume. The biodistribution were expressed as %ID/mL, which represents the percentage injected dose per milliliter of tissue.

### ^18^F-FDG PET/CT imaging

For the pancreatic cancer xenograft-bearing nude mice, ^18^F-FDG PET/CT imaging was performed before and after treatment (n = 3 per group). Mice were fasted for 8 h and then were administered with 7.4 MBq of ^18^F-FDG via the tail vein. About 1 h later, PET/CT imaging was carried out. Maximum standardized uptake value (SUV_max_) was calculated by the TrueD system automatically by drawing ROI.

### Statistical analysis

Data are presented as the mean ± SD. Analysis of variance (ANOVA) and Student’s *t*-test were used for the comparisons among groups. P value less than 0.05 was considered statistically significant. All experiments were performed in triplicate.

## Results

### Radiochemical characteristics of ^131^I-FAP-2286

**Fig. 1:**
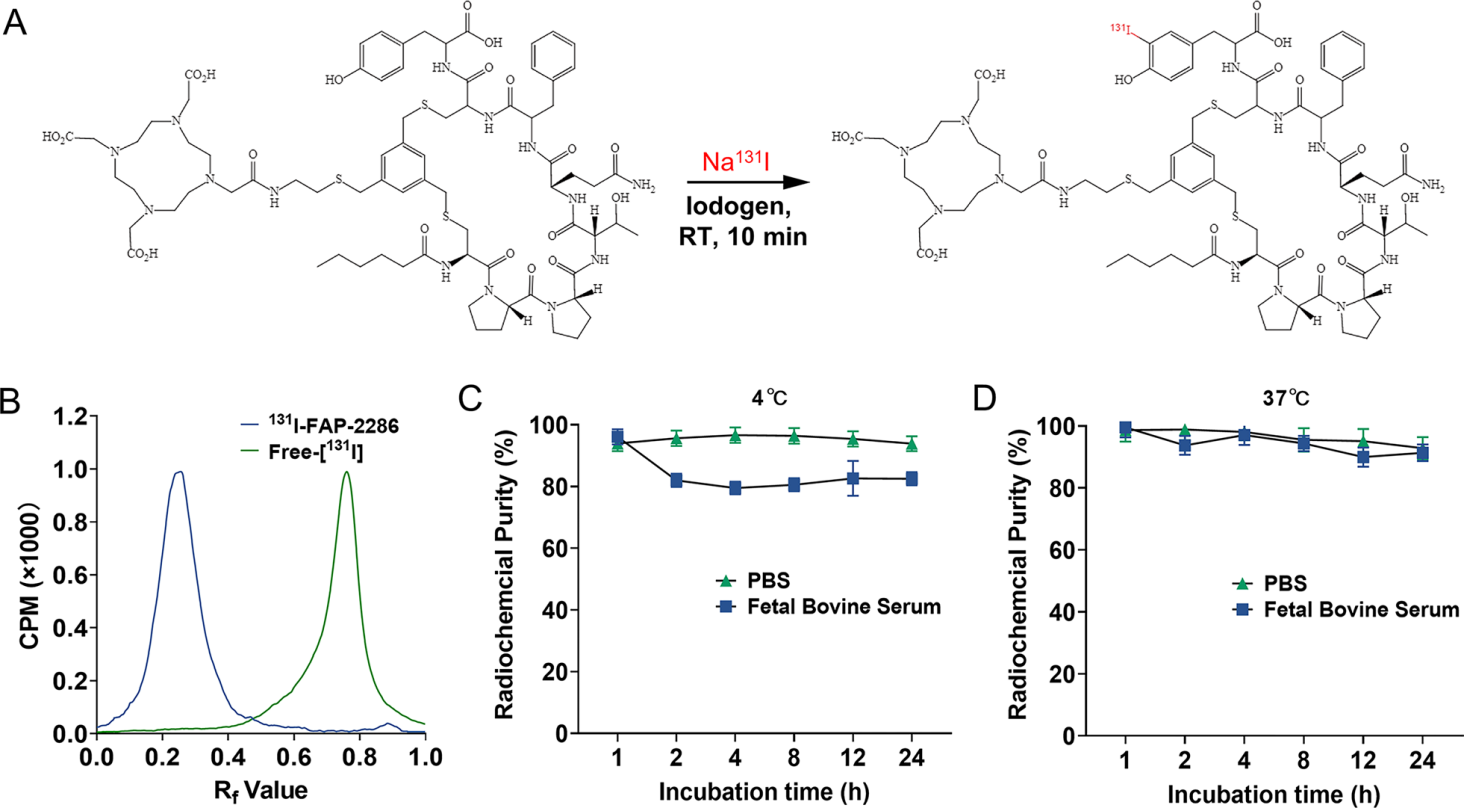
**A** Schematic presentation of labeling synthesis of ^131^I-FAP-2286. **B** Radio-iTLC spectrum of ^131^I-FAP-2286 and free-[^131^I]. The in vitro stability of ^131^I-FAP-2286 in PBS and 10% FBS solution at 4 ℃ (**C**) and 37 ℃ (**D**)

Figure 1A illustrates the labeling process of ^131^I-FAP-2286. The labeling rate of ^131^I-FAP-2286 was over 99% (Fig. 1B), allowing its use without purification. After a 24-h incubation with PBS or 10% FBS at 4 °C and 37 °C, the radiochemical purity of ^131^I-FAP-2286 remained > 80% (Fig. 1C), meaning that ^131^I-FAP-2286 has a good in vitro stability.

### ^131^I-FAP-2286 exposure induces autophagy in PANC-1 cells

**Fig. 2:**
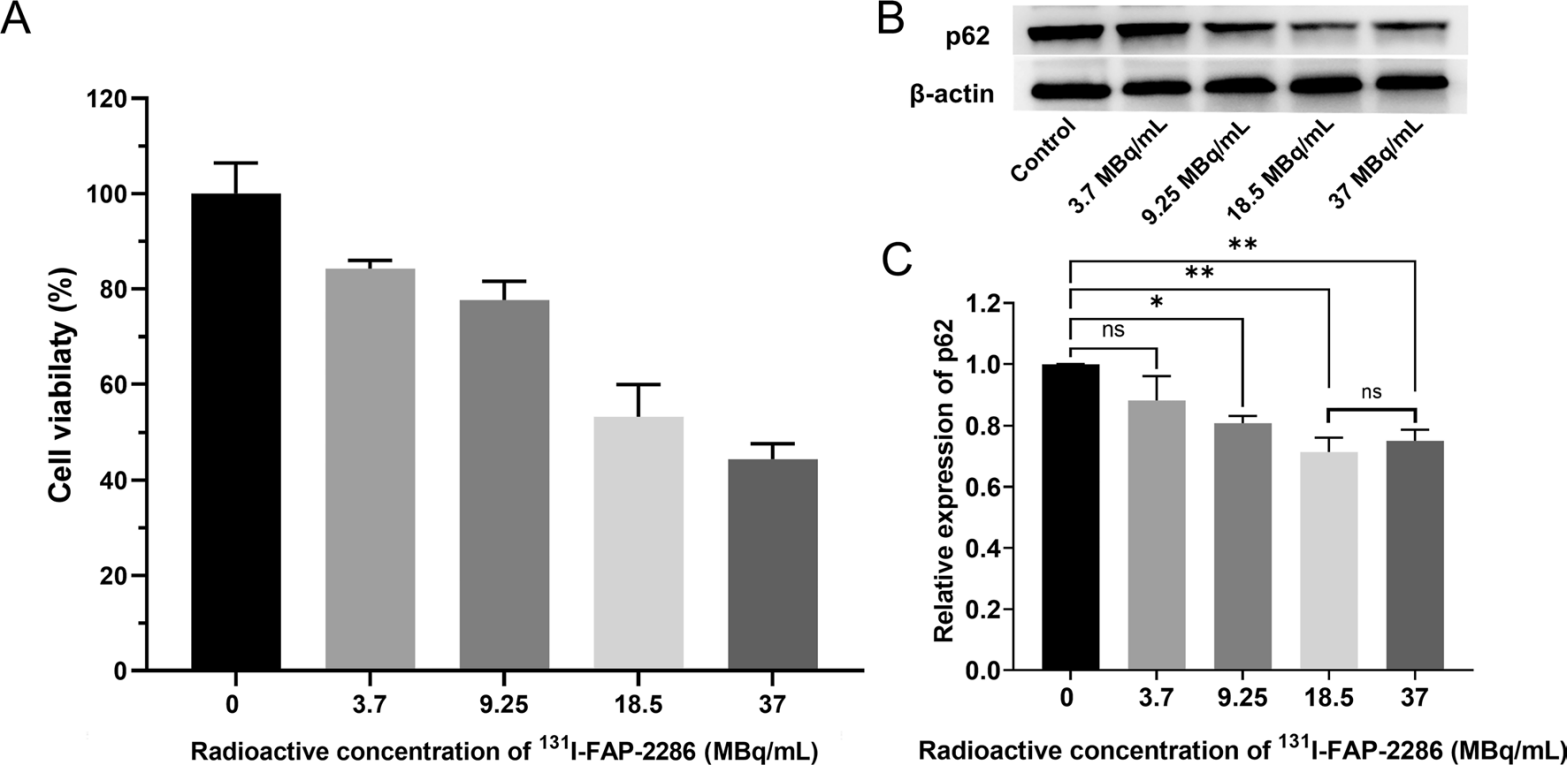
**A** The viability of PANC-1 cells treated with various radioactivity concentrations of ^131^I-FAP-2286. **B**, **C** Representative western blot images and semi-quantification analysis of p62 expression in PANC-1 cells. ns: not statistically significant, *P < 0.05, **P < 0.01

^131^I-FAP-2286 exposure induced the proliferation inhibition of PANC-1 cells in a radioactive dose-dependent manner (Fig. 2A). P62 is a well-established autophagy marker, whose expression is inversely correlation with the autophagy level. As shown in Fig. 2B, C, the expression of p62 in PANC-1 cells was reduced gradually along with the radioactive concentration increasement of ^131^I-FAP-2286, indicating that the cell autophagy level was elevated. Additionally, under the condition of indirect coculture with CAFs, p62 expression in PANC-1 cells was further decreased after ^131^I-FAP-2286 exposure (Additional file 1: Fig. S1), meaning that CAFs enhanced the radiation-induced autophagy of PANC-1 cells. Notably, 9.25 MBq/mL of ^131^I-FAP-2286 exhibited mild cytotoxicity, but produced obvious autophagic effect on PANC-1 cells. Thus, 9.25 MBq/mL as a representative concentration was chosen for the subsequent experiments.

### Autophagy inhibitor reverses the elevated autophagy level in PANC-1 cells induced by ^131^I-FAP-2286

**Fig. 3:**
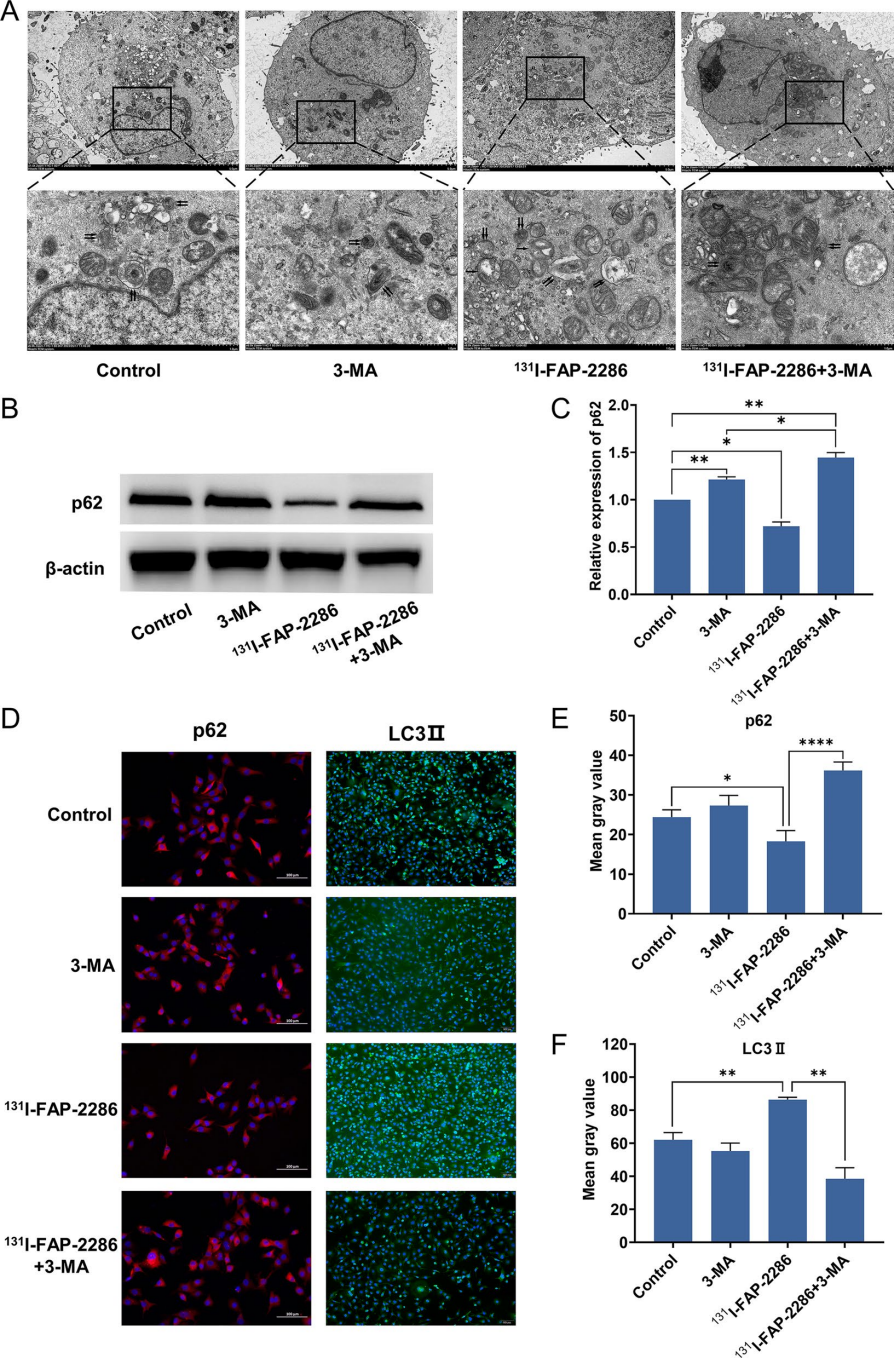
**A** Representative TEM images of autophagosome (single black arrow) and autophagolysosome (double black arrow) in the cytoplasm of PANC-1. **B**, **C** Representative western blot images and semi-quantification analysis of p62 expression in PANC-1 cells. **D**–**F** Representative fluorescent images of p62 (red) and LC3II punctuation (green) in PANC-1 cells, and the quantification analysis of mean fluorescence intensity per cell. Nuclei were stained with DAPI (blue). *P < 0.05, **P < 0.01, ****P < 0.0001

TEM images revealed that more cytoplasmic autophagosomes appeared in ^131^I-FAP-2286-treated PANC-1 cells compared to controls, but this phenomenon was reversed by the autophagy inhibitor 3-MA (Fig. 3A). Western blot experiments shown that, in^131^I-FAP-2286 treatment group, the expression of p62 was lowered, but was restored by the application of 3-MA (Fig. 3B, C). Consistently, the average p62 fluorescence intensity per cell decreased significantly in PANC-1 cells treated with ^131^I-FAP-2286, but recovered in ^131^I-FAP-2286 + 3-MA group (Fig. 3D and E). LC3II is another autophagy marker, whose expression is positively correlated with autophagy level. As shown in Fig. 3D and F, the mean fluorescence intensity per cell of LC3II increased significantly in ^131^I-FAP-2286 group, but the addition of 3-MA can efficiently block this effect. Together, the above results indicated that 3-MA inhibited the elevated autophagy of PANC-1 cells induced by ^131^I-FAP-2286.

### Autophagy inhibitor promotes the apoptosis of PANC-1 cells induced by ^131^I-FAP-2286

**Fig. 4:**
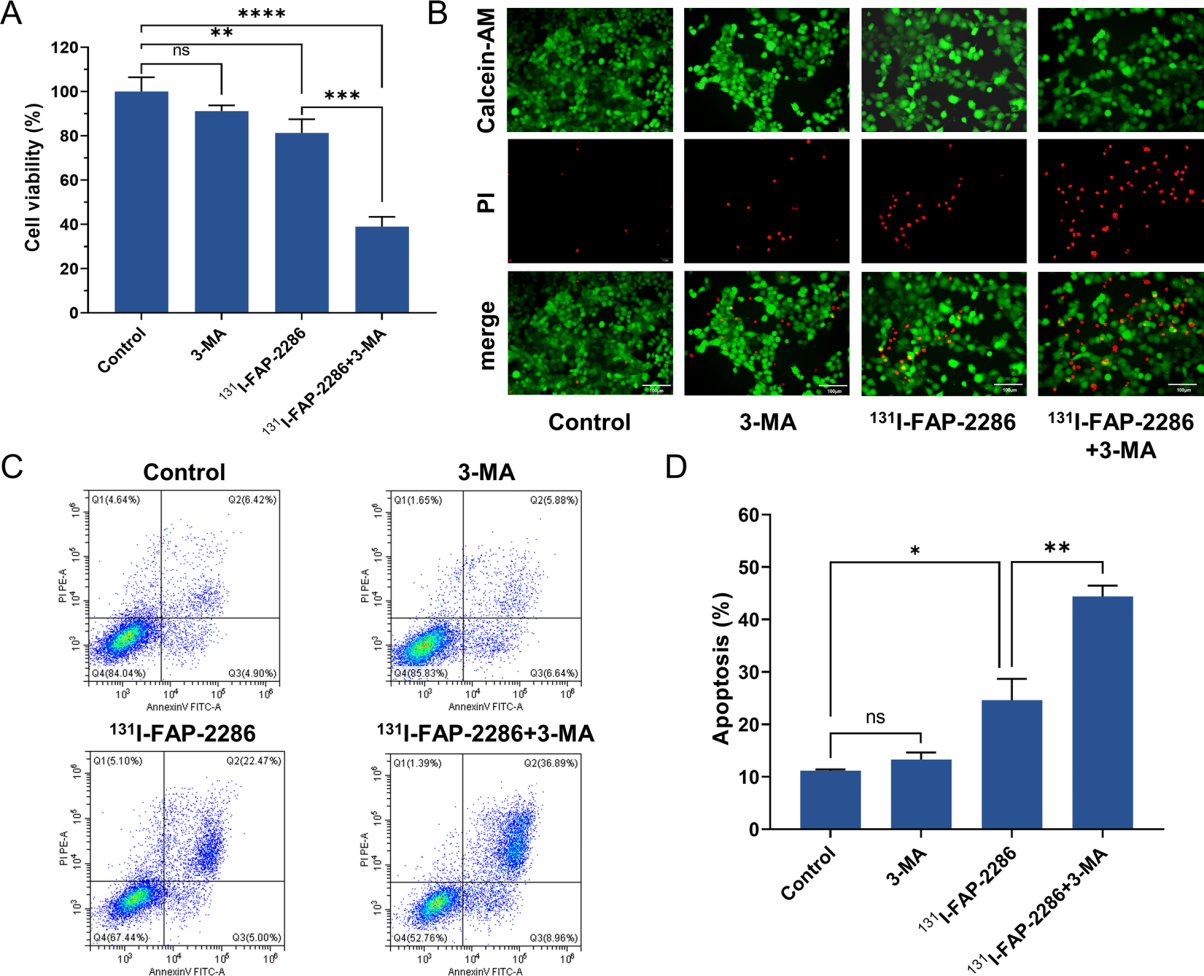
**A** The cell viability of PANC-1 cells in different groups. **B** Representative Calcein-AM/PI staining fluorescence images of PANC-1 cells. Scale bar: 100 μm. **C**, **D** Annexin V-FITC/PI apoptosis analysis of PANC-1 cells with different treatments. ns: not statistically significant, *P < 0.05, **P < 0.01, ***P < 0.001, ****P < 0.0001

As shown in Fig. 4A, 3-MA treatment alone did not influence the viability of PANC-1 cells. However, ^131^I-FAP-2286 significantly inhibited the proliferation of PANC-1 cells, and this effect was further amplified by 3-MA. Calcein-AM/PI staining results showed that ^131^I-FAP-2286 combined with 3-MA treatment induced more PANC-1 cells death compared to monotherapy group (Fig. 4B). This result was further confirmed by the apoptosis detection, which showed that 3-MA promoted ^131^I-FAP-2286-induced PANC-1 cells apoptosis (Fig. 4C, D). Notably, 3-MA treatment alone did not cause obvious apoptosis. Taken together, these results demonstrated that autophagy inhibitor 3-MA enhanced ^131^I-FAP-2286-triggered apoptosis in PANC-1 cells.

### The biodistribution of ^131^I-FAP-2286 in pancreatic cancer xenograft-bearing nude mice

**Fig. 5:**
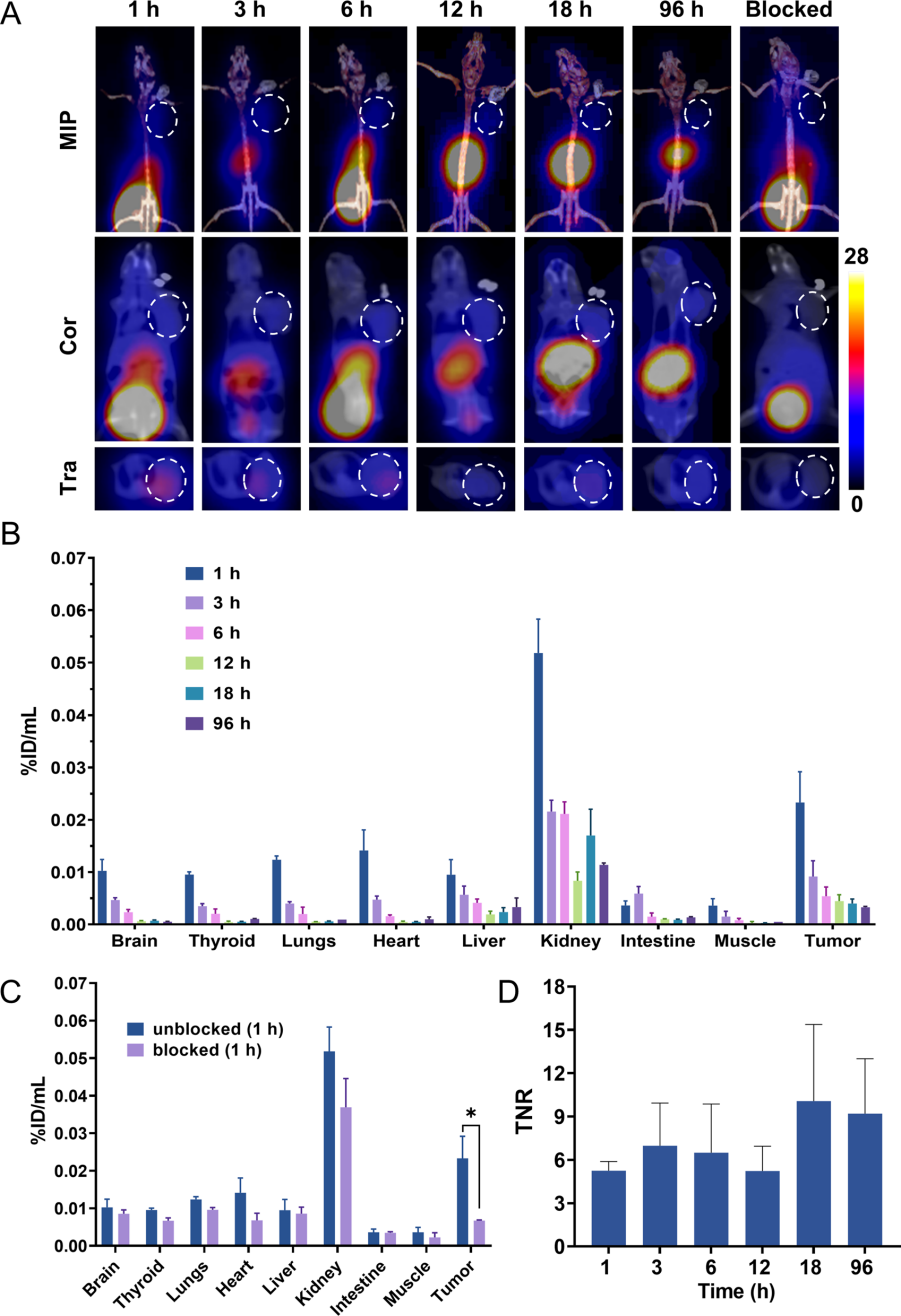
**A** Representative maximum intensity projection (MIP), coronal (Cor) and transverse (Tra) images of ^131^I-FAP-2286 SPECT/CT at different time points in pancreatic cancer xenograft-bearing nude mice. The dotted circle points the location of the tumor. **B**, **C** ^131^I-FAP-2286 uptake of different organs in the unblocking group and blocking group. **D** TNR of ^131^I-FAP-2286 at different time points. *P < 0.05

SPECT/CT imaging demonstrated a significant uptake and retention of ^131^I-FAP-2286 in tumor lesions up to 96 h post injection (Fig. 5A). No ^131^I-FAP-2286 uptake was observed in mice bearing-PANC-1 alone tumors, and free-[^131^I] did not accumulate in tumors with the co-injection of PANC-1 and CAFs (Additional file 1: Figs. S2, S3). As shown in Fig. 5B, ^131^I-FAP-2286 was mainly concentrated in the kidney and liver at 1 h after intravenous injection, suggesting that ^131^I-FAP-2286 was primarily excreted through the urinary system. The radioactivity in the thyroid and stomach was low, meaning that ^131^I-FAP-2286 is relatively stable to avoid in vivo deiodination. In addition, the tumor uptake of ^131^I-FAP-2286 in the blocking group was significantly lower than that in the non-blocking group ((0.0067 ± 0.0001%ID/mL) vs (0.0233 ± 0.0026%ID/mL), *P* = 0.032) (Fig. 5C). According to the target-to-normal tissue ratio (TNR), the maximum value occurred at 18 h post-injection of ^131^I-FAP-2286 (Fig. 5D). Notably, compared to the tumor with PANC-1 injection alone, the tumor with the co-injection of PANC-1 and CAFs had a stronger positive expression of FAP and α-SMA (Additional file 1: Fig. S4), indicating that it was CAFs that provided the target for ^131^I-FAP-2286.

### Autophagy inhibition enhances the TRT efficacy of ^131^I-FAP-2286 in pancreatic cancer

**Fig. 6:**
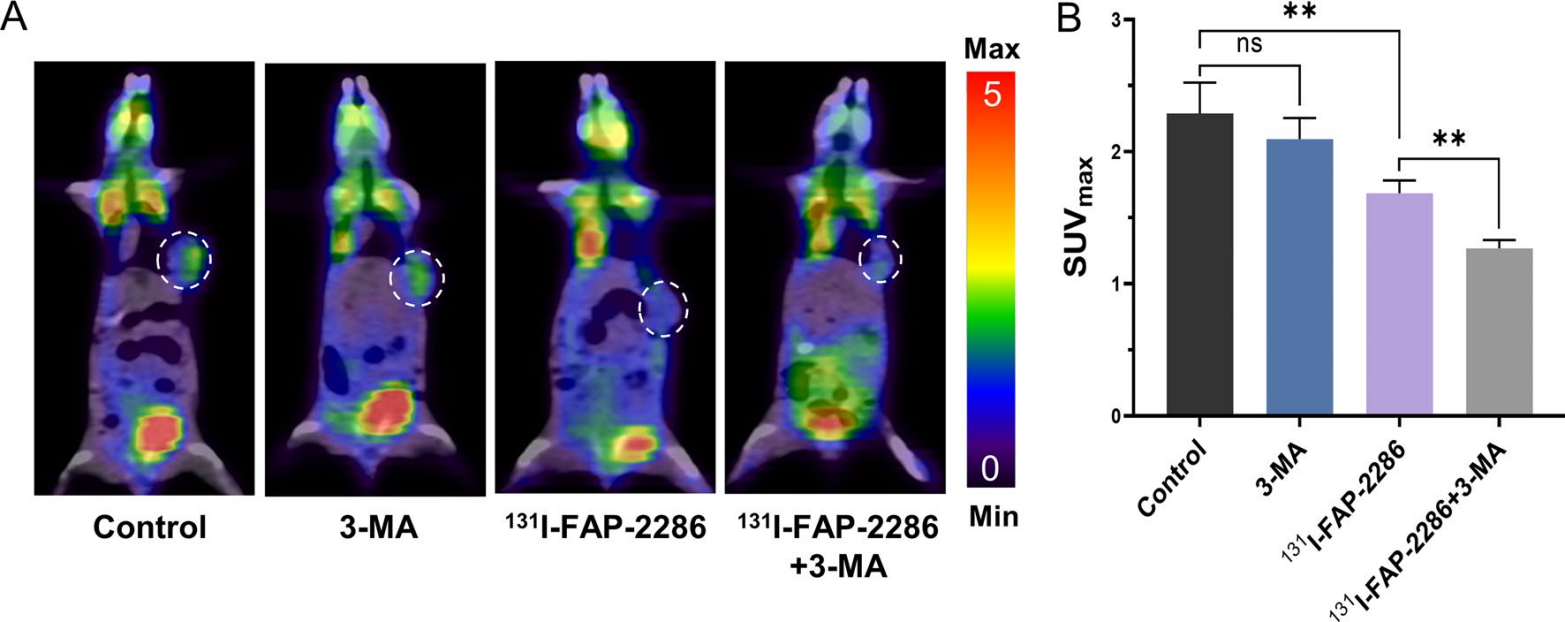
Representative ^18^F-FDG PET/CT images (**A**) and the SUV_max_ of tumor (**B**) in pancreatic cancer xenograft-bearing nude mice with different treatments (control (saline), 3-MA, ^131^I-FAP-2286, ^131^I-FAP-2286 + 3-MA). The dotted circle indicates tumors. ns: not statistically significant, **P < 0.01

The ^18^F-FDG uptake reflects the activity of tumor. As shown in Fig. 6A, B, compared to control group, ^131^I-FAP-2286 TRT markedly reduced the uptake of ^18^F-FDG in tumor (SUV_max_: 2.29 ± 0.16 vs 1.68 ± 0.08, *P* < 0.01), but the lowest SUV_max_ of tumor was observed in ^131^I-FAP-2286 + 3-MA group. Besides, the SUV_max_ in 3-MA group was equivalent to the control group. These results indicated that ^131^I-FAP-2286 TRT was capable of curbing pancreatic tumor growth, and the addition of autophagy inhibitor 3-MA could enhance the therapeutic effect.

**Fig. 7:**
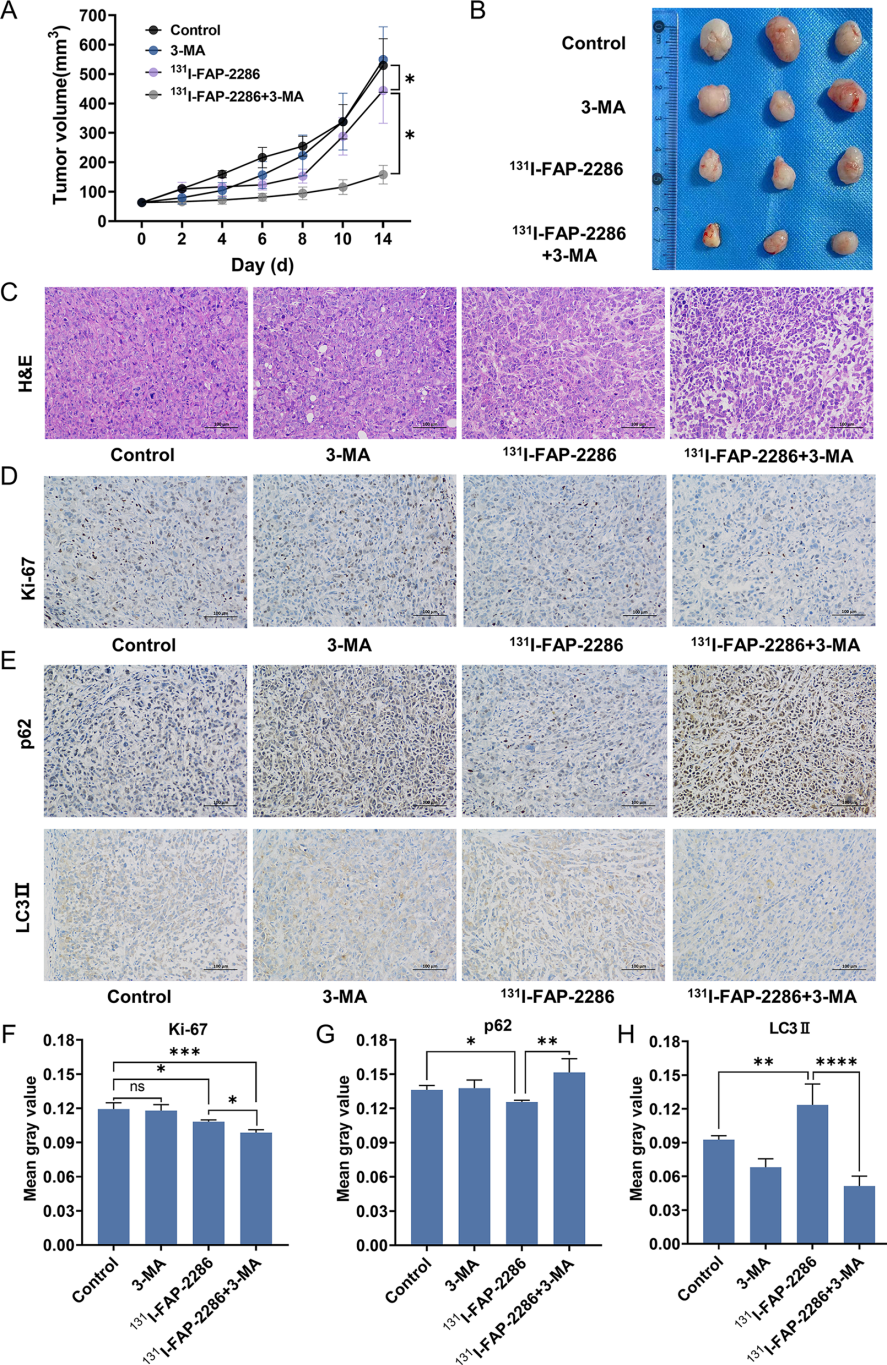
**A** Tumor growth curves of different treatment groups. **B** The photograph of the isolated tumors. **C** Representative H&E staining images of tumors tissues in different groups. **D**–**H** Immunohistochemistry images and quantification analysis of Ki-67, p62, LC3II expression in the tumor with different treatments. ns: not statistically significant, *P < 0.05, **P < 0.01, ***P < 0.001, ****P < 0.0001

Being consistent with the results of ^18^F-FDG PET/CT, ^131^I-FAP-2286 TRT significantly inhibited the tumor growth, and 3-MA alone did not affect the tumor growth. Notably, the combination treatment of ^131^I-FAP-2286 and 3-MA displayed the strongest anti-tumor effect (Fig. 7A, B). In addition, H&E staining assay showed that ^131^I-FAP-2286 TRT increased the area of tumor necrosis and apoptosis, which was more serious in ^131^I-FAP-2286 + 3-MA group, but while 3-MA alone did not cause this effect (Fig. 7C). Ki-67 is frequently used to assess the proliferation of tumor cells. As shown in Fig. 7D and F, after ^131^I-FAP-2286 TRT, the expression of Ki-67 was obviously down-regulated, meaning that the proliferation of tumor cells was suppressed. It should be noted that, compared to ^131^I-FAP-2286 TRT alone, the combination treatment group possessed lower ki-67 expression, signifying better therapeutic effect.

The autophagy level of tumors with different treatment also was detected. ^131^I-FAP-2286 TRT decreased p62 expression but increased LC3II expression of tumor, confirming that ^131^I-FAP-2286 exposure also induced autophagy in vivo. In ^131^I-FAP-2286 + 3-MA group, the TRT-mediated autophagy of pancreatic tumors was restored (Fig. 7E, G, H). The above results suggested that the enhanced anti-tumor effect of the combination treatment of ^131^I-FAP-2286 and 3-MA could be attributed to the inhibition of autophagy.

### Safety of ^131^I-FAP-2286 + 3-MA treatment

**Fig. 8:**
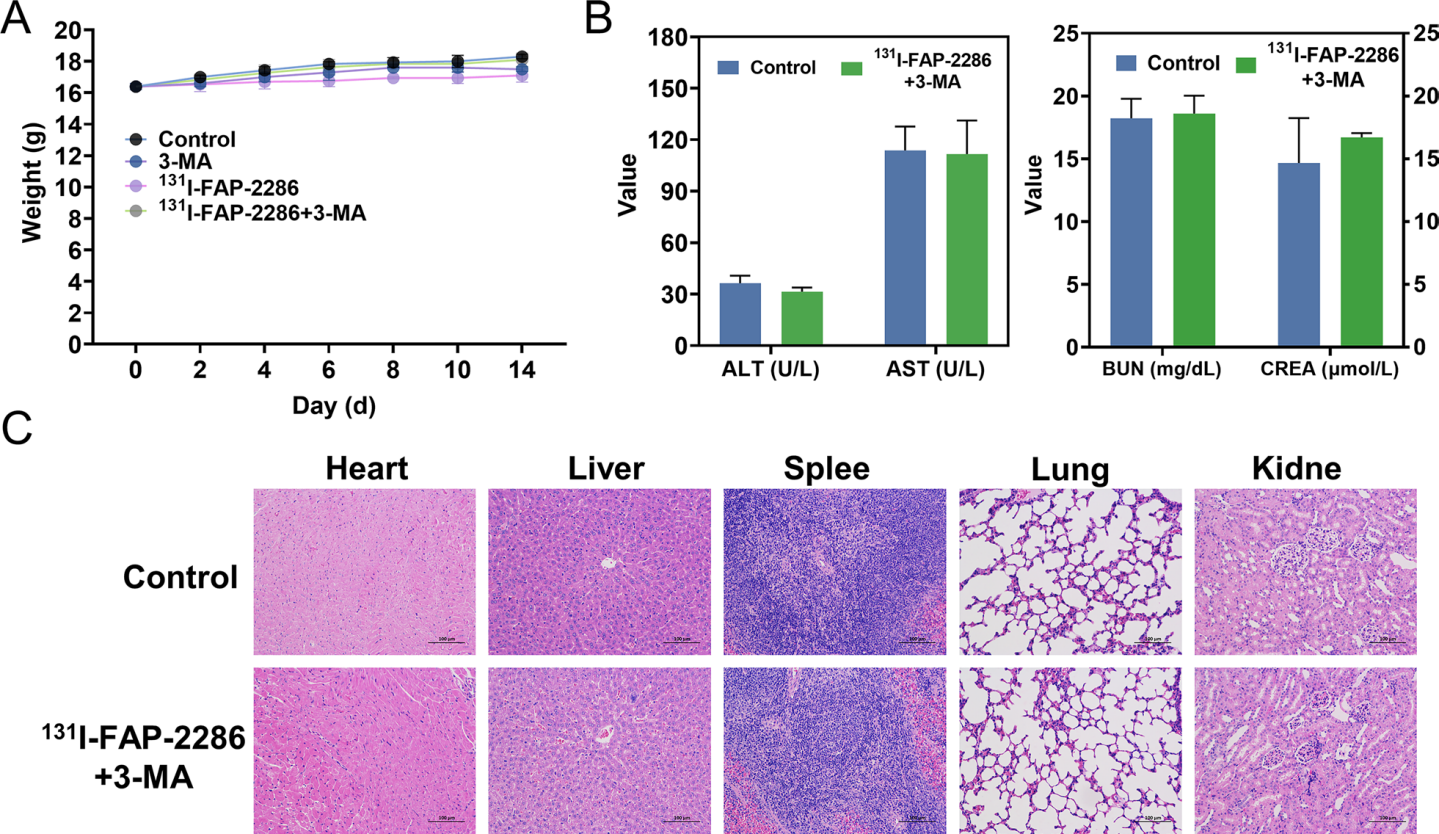
The in vivo toxicity study of ^131^I-FAP-2286 + 3-MA. **A** Body weight change of mice after different treatments. **B** ALT, AST, BUN and CREA level in the serum of mice at 2 weeks after injection of saline (control) and ^131^I-FAP-2286 + 3-MA. **C** H&E-stained images of major organs including heart, liver, spleen, lung and kidney collected from the control mice and ^131^I-FAP-2286 + 3-MA injected mice, respectively

After treatment, no abnormalities were found in mice weight, hepatorenal functions and creatine kinase (Fig. 8A, B). Besides, H&E sections of the heart, liver, spleen, lung and kidney indicated no established systemic biological toxicity upon ^131^I-FAP-2286 + 3-MA therapy (Fig. 8C).

## Discussion

**Fig. 9:**
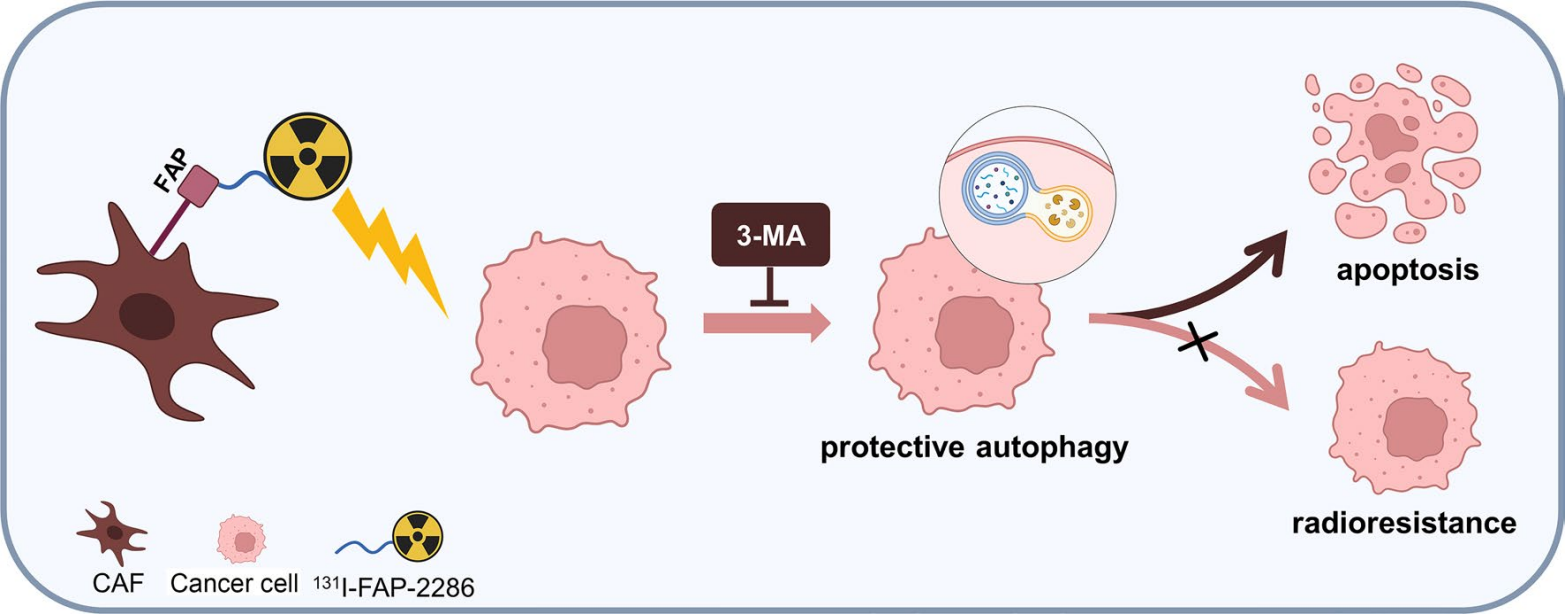
CAFs-targeted ^131^I-FAP-2286 induces the protective autophagy of pancreatic cancer cells, limiting the therapeutic effect of TRT, and the addition of 3-MA promotes cancer cell apoptosis by inhibiting autophagy (Created with BioRender.com)

Our present study suggested that ^131^I-FAP-2286 exposure can induce PANC-1 cells proliferation inhibition and autophagy, and the addition of the autophagy inhibitor 3-MA promoted the in vitro anti-tumor effect of ^131^I-FAP-2286 through restraining autophagy. In pancreatic cancer xenograft-bearing nude mice, ^131^I-FAP-2286 can efficiently target the tumor and exert TRT effect along with the autophagy induction. The combination of 3-MA and ^131^I-FAP-2286 achieved a better therapeutic effect, which was attributed mainly to the destruction of the protective autophagy of tumor (Fig. 9).

As an effective radiotherapy approach, TRT has demonstrated considerable promise in various types of cancer, including pancreatic cancer [22, 23]. For the TRT of pancreatic cancer, the specificity of pancreatic cancer biomarkers, such as FAP, is conducive to the improvement of the radiopharmaceutical uptake ratio of tumor-to-normal tissue [24–27]. FAP-2286 has an advantage of accumulating in the FAP-positive tumor tissues for a relative long time [28, 29]. ^131^I-FAP-2286 can specifically target CAFs in the tumor stroma, and further irradiates tumor cells via non-specific cross-irradiation [30, 31]. According to the immunohistochemistry assay, the expression of α-SMA, a well-known marker of CAFs, was consistent with FAP in the pancreatic cancer xenograft model with the co-injection of PANC-1 and CAFs, confirming the presence of abundant FAP-positive CAFs. Our results confirmed that ^131^I-FAP-2286 was rapidly cleared from non-target organs except for kidney and remained in pancreatic cancer xenografts more than 96 h, which was the important premise for the TRT. From the view point of the safety of TRT, using lower radioactive dose for better therapeutic effect is the everlasting pursuit. Therefore, validating the feasibility of ^131^I-FAP-2286 TRT for pancreatic cancer just is the first step.

Numerous studies have shown that radiation exposure can activate the protective autophagy of tumor cells, which may produce negative effect on the efficacy of radiotherapy [20, 32]. Radiotherapy induced ROS-dependent autophagy in macrophages through unfolded protein response [33]. ^131^I-FAP-2286 TRT may also face a similar situation. Our results demonstrated that ^131^I-FAP-2286 reduced p62 expression and promoted the formation of autophagosomes, meaning the occurrence of the protective autophagy. Within a certain dose range, ^131^I-FAP-2286 exposure induced autophagy in a dose-dependent manner. Autophagy inhibitor 3-MA blocks the early process of radiation-induced autophagy by impairing the formation of the PI3K complex [34, 35]. Furthermore, it has been reported that 3-MA can induce caspase-dependent cell death without the aid of autophagy inhibition [36]. When the autophagy was inhibited by 3-MA, ^131^I-FAP-2286 displayed a stronger anti-tumor ability, suggesting that blocking the autophagy activation of pancreatic cancer cells decrease their ability to resist radiation damage. It has been reported that autophagy inhibition has the potential to enhance the radio-sensitivity of radiation-resistant pancreatic cancer [37, 38]. According to our present result, suppression of autophagy has a promotional effect on ^131^I-FAP-2286 TRT, and the combination of autophagy inhibitor and TRT is expected to make for better outcomes via inducing multiple types of tumor cell death simultaneously, offering a unique approach to enhance the efficacy of radiotherapy [39].

Studies have shown that tumor cells can enter a senescent or dormant state, and further become resistant to radiotherapy [40, 41]. Autophagy inhibition has the ability of destroying the senescence and dormancy of tumor cells, which also may be a potential mechanism of improving the therapeutic efficacy. The future research will focus on elucidating the underlying molecular mechanisms of TRT-induced autophagy, as well as the synergistic effect of TRT in combination with autophagy inhibition. Our present findings provide a reference for the preclinical study of ^131^I-FAP-2286 TRT combined with autophagy inhibitors in the FAP-expressed tumors.

In conclusion, our study suggests that autophagy inhibition can significantly improve the TRT efficacy of ^131^I-FAP-2286 in pancreatic cancer, providing a novel strategy for pancreatic cancer radiation therapy.

### Supplementary Information

**Additional file 1: Figure S1.** (A-B) Representative western blot image and the semi-quantification analysis of p62 expression in PANC-1 cells. I, Control; II, ^131^I-FAP-2286; III, ^131^I-FAP-2286 + indirect coculture with CAFs (1: 1); IV, ^131^I-FAP-2286 + indirect coculture with CAFs (1: 5). *P < 0.05, ***P < 0.001, ****P < 0.0001. **Figure S2.** Representative coronal (Cor) and transverse (Tra) images of ^131^I-FAP-2286 SPECT/CT at different time points in only-PANC-1 tumor xenograft-bearing nude mice**.** The dotted circle points the location of the tumor. **Figure S3.** Representative coronal (Cor) and transverse (Tra) images of free ^131^I SPECT/CT at different time points in pancreatic cancer xenograft-bearing nude mice with the co-injection of PANC-1 + CAFs. The dotted circle points the location of the tumor. **Figure S4.** Immunohistochemistry images of FAP and α-SMA expression in the xenograft tumors with PANC-1 + CAFs (A) and PANC-1 alone (B).

## Data Availability

All data generated or analysed during this study are included in this published article.
