# Supplementary material for: Autophagy inhibition improves the targeted radionuclide therapy efficacy of 131I-FAP-2286 in pancreatic cancer xenografts
Source: J Transl Med. 2024 Feb 15;22:156. doi: 10.1186/s12967-024-04958-6 (PMC10870561; doi:10.1186/s12967-024-04958-6)
Supplement: Supplementary file 1 — Additional file 1: Figure S1. (A-B) Representative western blot image and the semi-quantification analysis of p62 expression in PANC-1 cells. I, Control; II, 131I-FAP-2286; III, 131I-FAP-2286 + indirect coculture with CAFs (1: 1); IV, 131I-FAP-2286 + indirect coculture with CAFs (1: 5). *P < 0.05, ***P < 0.001, ****P < 0.0001. Figure S2. Representative coronal (Cor) and transverse (Tra) images of 131I-FAP-2286 SPECT/CT at different time points in only-PANC-1 tumor xenograft-bearing nude mice. The dotted circle points the location of the tumor. Figure S3. Representative coronal (Cor) and transverse (Tra) images of free 131I SPECT/CT at different time points in pancreatic cancer xenograft-bearing nude mice with the co-injection of PANC-1 + CAFs. The dotted circle points the location of the tumor. Figure S4. Immunohistochemistry images of FAP and α-SMA expression in the xenograft tumors with PANC-1 + CAFs (A) and PANC-1 alone (B). [file 12967_2024_4958_MOESM1_ESM.docx]

**Additional file**

**Autophagy inhibition improves** **the targeted radionuclide therapy efficacy of** **^131^I-FAP-2286 in** **pancreatic cancer xenografts**

Xingyu Liu^1,2,3^, Danni Li^2^, Tianbao Ma^2^, Xiu Luo^2^, Ye Peng^2^, Tao Wang^2^*, Changjing Zuo^2^*, Jianming Cai^1,3^*

*^1^ School of Public Health and Management, Wenzhou Medical University, Wenzhou 325035, Zhejiang, China.*

*^2^ Department of Nuclear Medicine, the First Affiliated Hospital of Naval Medical University, Shanghai 200433, China.*

*^3^ Department of Radiation Medicine, Faculty of Naval Medicine, Naval Medical University, Shanghai 200433, China.*

**^*^Corresponding authors:**

Tao Wang (e-mail: wangtao2086@smmu.edu.cn; Phone +86-021-31162247),

Changjing Zuo (e-mail: cjzuo@smmu.edu.cn; Phone +86-021-31162247),

Jianming Cai (e-mail: cjm882003@163.com; Phone +86-021-81871153).


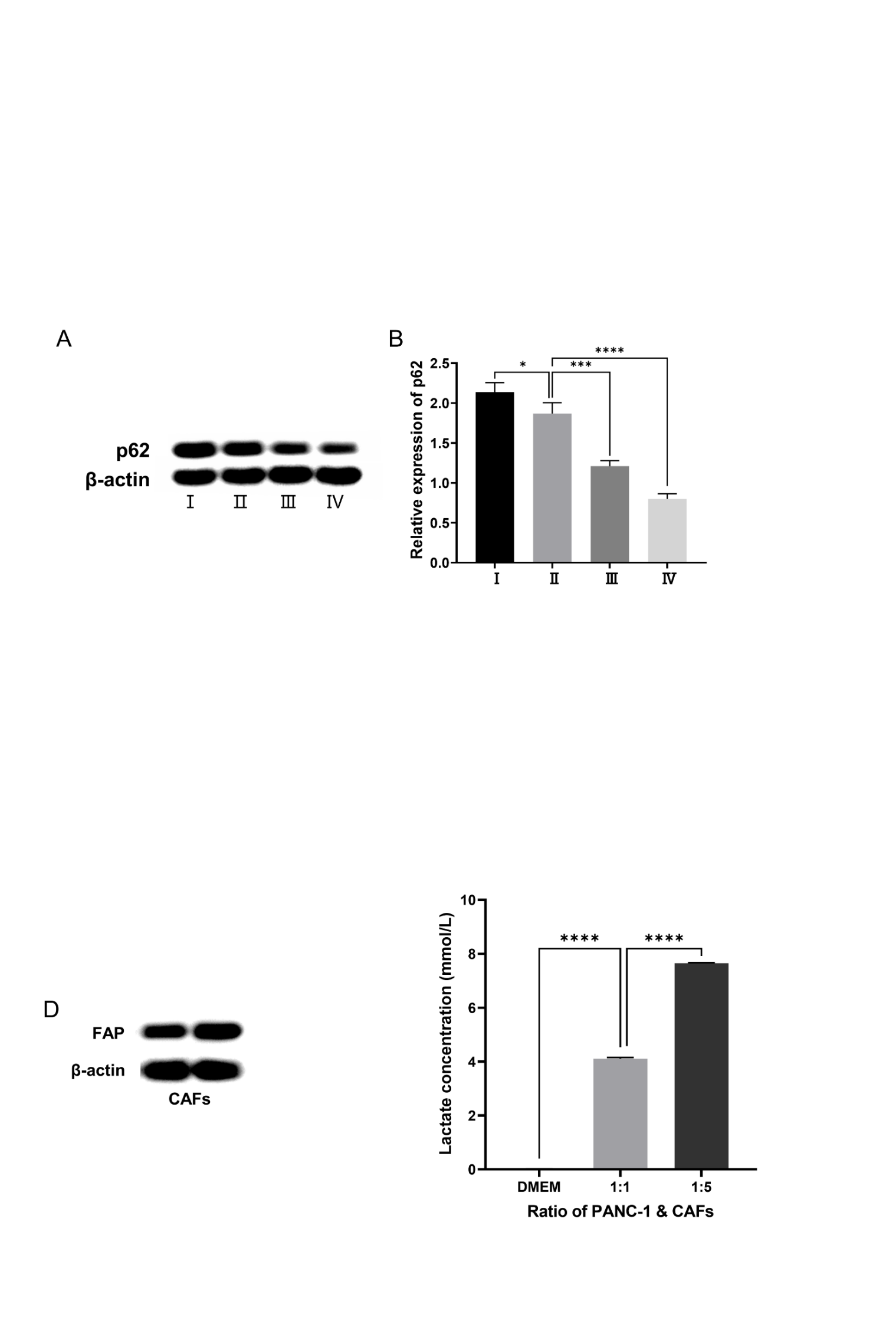


**Figure S1.** (A-B) Representative western blot image and the semi-quantification analysis of p62 expression in PANC-1 cells. Ⅰ, Control; Ⅱ, ^131^I-FAP-2286; Ⅲ, ^131^I-FAP-2286 + indirect coculture with CAFs (1 : 1); Ⅳ, ^131^I-FAP-2286 + indirect coculture with CAFs (1 : 5). **P* < 0.05, ****P* < 0.001, *****P* < 0.0001.

**
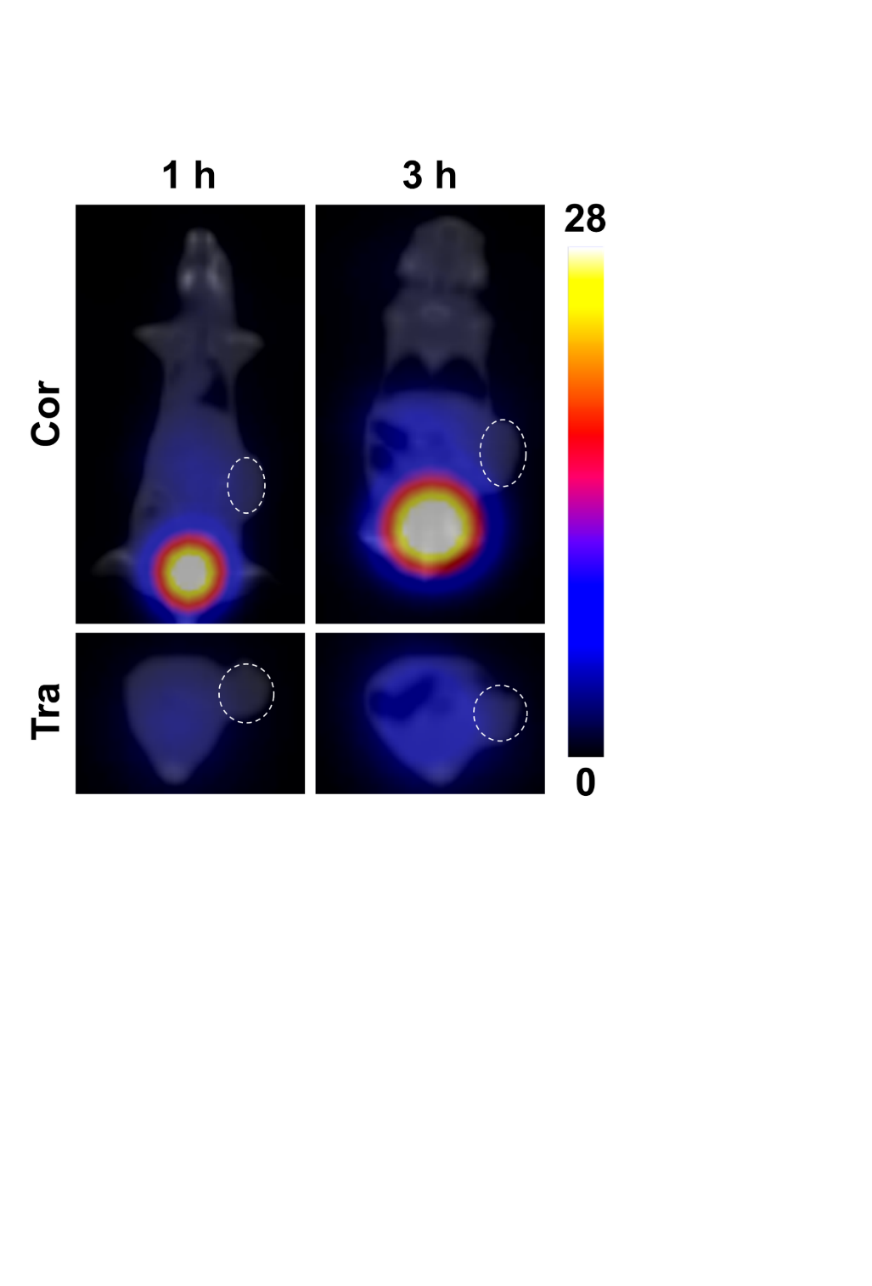
**

**Figure S2.** Representative coronal (Cor) and transverse (Tra) images of ^131^I-FAP-2286 SPECT/CT at different time points in only-PANC-1 tumor xenograft-bearing nude mice**.** The dotted circle points the location of the tumor.


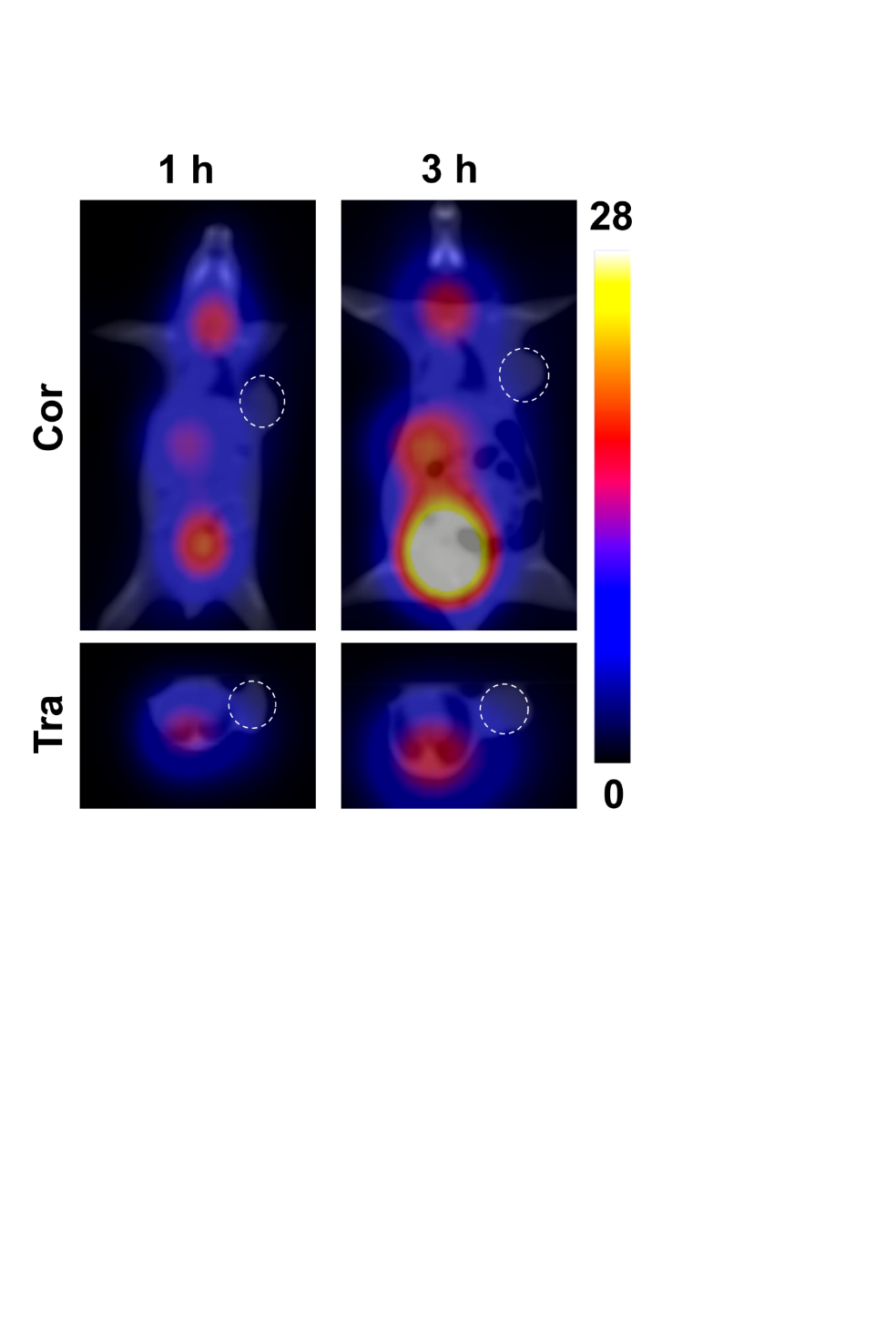


**Figure S3.** Representative coronal (Cor) and transverse (Tra) images of free ^131^I SPECT/CT at different time points in pancreatic cancer xenograft-bearing nude mice with the co-injection of PANC-1 + CAFs. The dotted circle points the location of the tumor.

**
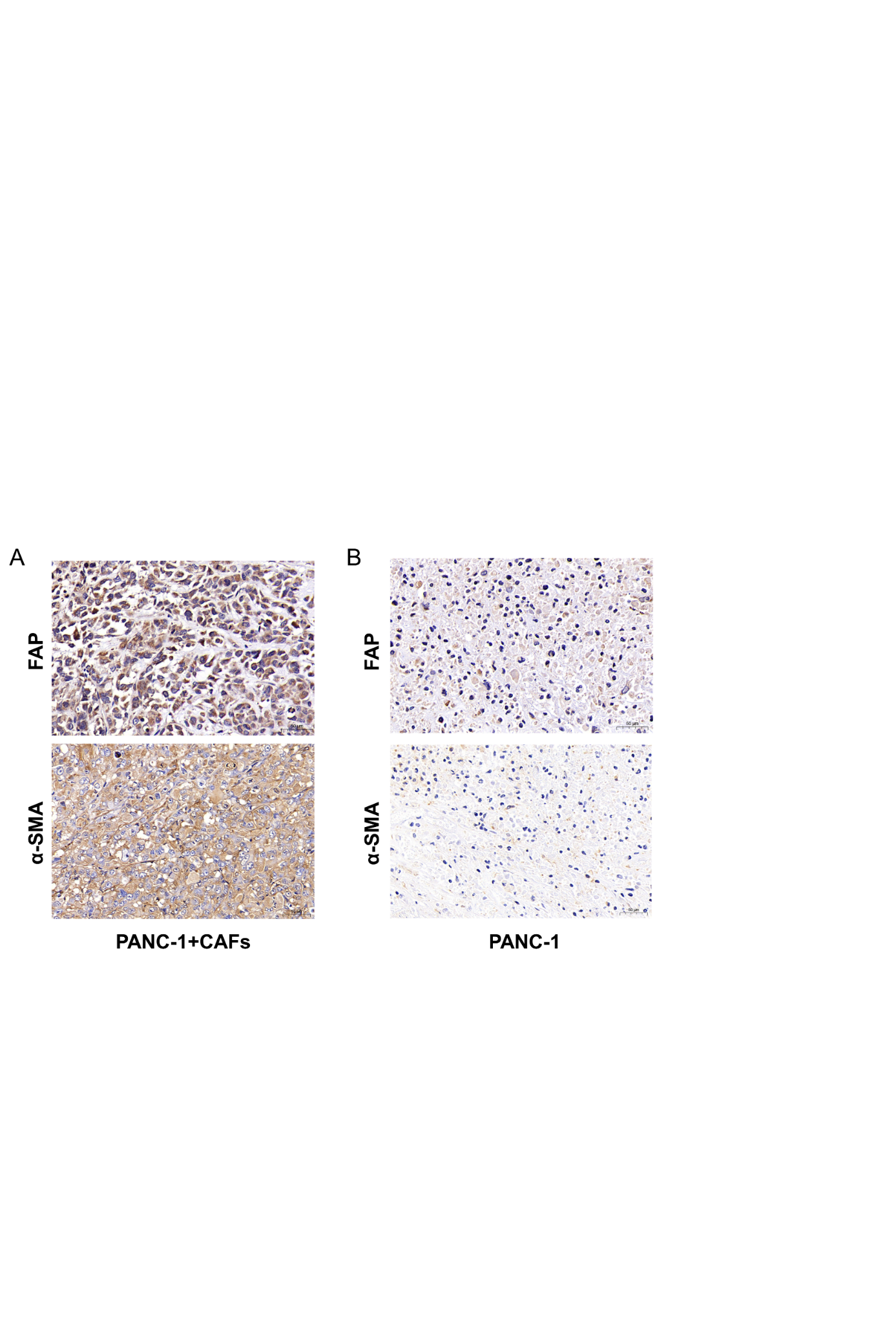
**

**Figure S4.** Immunohistochemistry images of FAP and α-SMA expression in the xenograft tumors with PANC-1 + CAFs (A) and PANC-1 alone (B).
